# Understanding Aquaporin Transport System in Eelgrass (*Zostera marina* L.), an Aquatic Plant Species

**DOI:** 10.3389/fpls.2017.01334

**Published:** 2017-08-03

**Authors:** S. M. Shivaraj, Rupesh Deshmukh, Javaid A. Bhat, Humira Sonah, Richard R. Bélanger

**Affiliations:** ^1^National Research Centre on Plant Biotechnology New Delhi, India; ^2^Département de Phytologie–Faculté des Sciences de l’Agriculture et de l’Alimentation, Université Laval, Québec QC, Canada; ^3^Department of Genetics and Plant Breeding, The Indian Agricultural Research Institute New Delhi, India

**Keywords:** solute transport, aquaporin evolution, comparative genomics, nodulin 26-like intrinsic proteins, silicon transporter

## Abstract

Aquaporins (AQPs) are a class of integral membrane proteins involved in the transport of water and many other small solutes. The AQPs have been extensively studied in many land species obtaining water and nutrients from the soil, but their distribution and evolution have never been investigated in aquatic plant species, where solute assimilation is mostly through the leaves. In this regard, identification of AQPs in the genome of *Zostera marina* L. (eelgrass), an aquatic ecological model species could reveal important differences underlying solute uptake between land and aquatic species. In the present study, genome-wide analysis led to the identification of 25 AQPs belonging to four subfamilies, plasma membrane intrinsic proteins (PIPs), tonoplast intrinsic proteins (TIPs), nodulin 26-like intrinsic proteins (NIPs), small basic intrinsic proteins (SIPs) in eelgrass. As in other monocots, the XIP subfamily was found to be absent from the eelgrass genome. Further classification of subfamilies revealed a unique distribution pattern, namely the loss of the NIP2 (NIP-III) subgroup, which is known for silicon (Si) transport activity and ubiquitously present in monocot species. This finding has great importance, since the eelgrass population stability in natural niche is reported to be associated with Si concentrations in water. In addition, analysis of available RNA-seq data showed evidence of expression in 24 out of the 25 AQPs across four different tissues such as root, vegetative tissue, male flower and female flower. In contrast to land plants, higher expression of PIPs was observed in shoot compared to root tissues. This is likely explained by the unique plant architecture of eelgrass where most of the nutrients and water are absorbed by shoot rather than root tissues. Similarly, higher expression of the TIP1 and TIP5 families was observed specifically in male flowers suggesting a role in pollen maturation. This genome-wide analysis of AQP distribution, evolution and expression dynamics can find relevance in understanding the adaptation of aquatic and land species to their respective environments.

## Introduction

Seagrasses are a group of monocotyledonous angiosperms that diverged from the terrestrial monocots about 130 MYA and subsequently adapted to completely submerged conditions of the marine environment (Janssen and Bremer, 2004). Eelgrass (*Zostera marina* L.) is an important aquatic weed found in the Atlantic and Pacific oceans as far as the Arctic circle. It provides habitat for several species of fish and invertebrates. Eelgrass improves water quality by absorbing pollutants, and prevents erosion by binding sediments (Moore, 2004). Considering its structural and functional role and importance in many coastal ecosystems, it was recently fully sequenced (Olsen et al., 2016). Genome analysis revealed loss and gain of multiple genes in *Z. marina* compared to terrestrial or floating aquatic plants, changes assumed to facilitate its adaptation to marine life (Olsen et al., 2016). These adaptations include morphological, physiological and breeding pattern modifications along with the ability to tolerate high salt levels of marine environments.

Notwithstanding the benefits it provides to marine ecosystems, worldwide estimation of eelgrass population suggests a 30% reduction over the past 30 years (Waycott et al., 2009). This is mostly associated with human disturbances (overfishing, eutrophication) and climate-change factors such as increased temperature, changes in mean sea level and biochemical composition of the sea water (Jarvis et al., 2014; Thom et al., 2014). Lower levels of silicon (Si) in marine water is also considered as one of the main reasons for reduced eelgrass population (Kamermans et al., 1999; Disney et al., 2014). In this context, a better understanding of the molecular mechanisms involved in the transport/acquisition of Si and other solutes in eelgrass could find relevance in explaining the alarming reduction in eelgrass populations.

The adverse effect of lower concentrations of dissolved Si on the growth of diatoms has been frequently reported. The diatoms are an important component of ecosystems, and changes in diatom populations substantially affect the marine food web (Ragueneau et al., 2006). Significant changes in the biogeochemistry and aquatic food webs of coastal marine environments have been observed with reduction in dissolved Si content in the Black and Baltic Seas (Humborg et al., 2000). A decreasing proportion of diatoms associated with an increase of flagellates was one of the interesting observations thought to explain the reduction in dissolved Si. The role of Si in aquatic ecosystems has been mostly studied with observational methods, so molecular experiments would contribute to a better definition of this role (Schoelynck and Struyf, 2016). More specifically, a better understanding of Si-transporting aquaporins in aquatic species would adequately support ecological experiments.

Aquaporins are small (21–34 kD) integral proteins, which form transmembrane channels to facilitate movement of water and many other solutes across the cell membrane. The topology of AQPs resembles that of an hourglass structure formed by six transmembranes (TM) α helices (H1 to H6) connected with five inter-helical loops (A to E). At the center of the pore formed by the six TM domains, two distinct constricts are formed, one with highly conserved NPA (Asn-Pro-Ala) motifs and another with four amino acid aromatic arginine (ar/R) region in the channel. These two constrictions determine solute permeability of the AQPs (Lee et al., 2005; Törnroth-Horsefield et al., 2006; Deshmukh et al., 2015).

Aquaporins are found in most living organisms including microbes, animals, and plants. However, AQP’s are comparatively more abundant and diverse in plants than in any other organisms. Based on sequence similarity, plant AQPs were grouped into five subfamilies: plasma membrane intrinsic proteins (PIPs), tonoplast intrinsic proteins (TIPs), nodulin 26-like intrinsic proteins (NIPs), small basic intrinsic proteins (SIPs) and uncategorized intrinsic protein (XIPs) (Chaumont et al., 2001; Johanson et al., 2001; Quigley et al., 2002; Kaldenhoff and Fischer, 2006). Among these, the PIP, TIP, and NIP subfamilies are well-characterized with regards to their localization and function. In addition to water, members of these subfamilies are known to transport urea (Li and Wang, 2014), lactic acid (Bienert et al., 2013), glycerol (Biela et al., 1999), metalloids like boron and silicon (Ampah-Korsah et al., 2016; Song et al., 2016; Ouellette et al., 2017), and gasses like ammonia (NH_3_) (Jahn et al., 2004), carbon dioxide (CO_2_) (Kaldenhoff et al., 2014) and hydrogen peroxide (H_2_O_2_) (Tian et al., 2016). All AQP subfamilies are widely distributed in different plant species including primitive land plants, with the exception of XIPs that are absent in some higher plants such as *Brassicaceae* and monocots (Gupta and Sankararamakrishnan, 2009; Deshmukh et al., 2015; Sonah et al., 2017). With the availability of complete genome sequences, the genes encoding AQPs have been characterized in many land plants such as *Arabidopsis*, rice, *Populus*, soybean, canola and tomato (Deshmukh et al., 2016a). Surprisingly enough, no systematic study of AQPs has been carried out in aquatic plant species. The recent availability of the annotated genome sequences of eelgrass provides an opportunity to investigate and compare the role of AQPs in plants adapted to aquatic environments.

In the present study, we have performed a genome-wide identification of AQPs in *Z. marina*. Subsequently, characterization of AQPs was conducted based on phylogenetic analysis, gene structure organization, conserved motifs, ar/R selectivity filters, and homology-based 3D protein structure. Finally, AQP expression profiling in different tissues was studied using available transcriptomic data.

## Materials and Methods

### Genome-Wide Identification and Distribution of AQPs in *Zostera marina*

The genome sequence of *Z. marina* V2.2 was retrieved from the Phytozome database^1^. A local database of the predicted protein sequences from *Z. marina* genome was created using BioEdit ver. 7.2.5 (Hall, 1999). Aquaporin homologs were identified by BLASTp search performed against the local database using query sequences of 141 AQPs from rice, *Arabidopsis*, and soybean (Supplementary Data Sheet 1). An *e*-value of 10^-5^ was kept as an initial cut-off to identify high scoring pairs (HSPs). The blast output was tabulated, and the HSPs with >100-bit score was selected. Finally, redundant hits were removed to select unique sequences for further analysis.

### Structural Characterization of *Zostera marina* Aquaporins

The genomic and cDNA sequences of AQPs identified in *Z. marina* were retrieved from Phytozome database. Structural annotations of the gene models (in gff3 format) were also retrieved from Phytozome. The gene structure of *Z. marina* AQPs was analyzed using Gene Structure Display Server (GSDS) ver. 2.0 (Hu et al., 2015).

### Identification of Functional Motif and Transmembrane Domains

The NPA motifs were identified in protein sequences using conserved domain database at NCBI (CDD). Aquaporins with missing NPA motifs were confirmed with a manual examination. Transmembrane domains in the genes were identified using TMHMM and SOSUI software tools^2^^,^^3^. The TM domains were manually evaluated to confirm alterations or complete loss.

### Phylogenetic Analysis of *Zostera marina* AQPs

The AQP sequences were aligned using CLUSTALW alignment function in MEGA6 (Kumar et al., 2008). The phylogenetic tree was constructed by using maximum likelihood method, and the stability of the branch node was measured by performing 1000 bootstraps. The subfamilies PIP, SIP, TIP, NIP, and XIPs were classified in accordance with the nomenclature used for *Arabidopsis*, rice and poplar (Quigley et al., 2002; Deshmukh et al., 2015).

### Expression Profiling of *Zostera marina* Aquaporins

The RNA-Seq dataset available at SRA database under the accession SRP056873 was used to analyze the expression of AQPs. A heat map for expression of AQPs was constructed using TIGR Multi Experiment Viewer (MeV^4^). Hierarchical clustering with average linkage method was performed to cluster the genes (Sonah et al., 2016).

## Results

### Genome-Wide Identification and Distribution of AQPs in *Zostera marina*

Genome-wide analysis of *Z. marina* led to the identification of 25 genes encoding AQPs (Supplementary Table 1). Conserved domain analysis confirmed candidate AQPs as members of the MIP (Major Intrinsic Protein) family (Supplementary Table 2). Prediction of transmembrane helices based on a hidden Markov model revealed the presence of six signature transmembrane domains in 21 out of the 25 identified AQPs (Supplementary Table 3). Furthermore, homology based tertiary protein structure of the AQPs confirmed the typical hourglass-like structure for all 25 proteins.

The *Z. marina* AQPs were found to be distributed among 22 scaffolds. Out of the 22 scaffolds, 19 contained only one AQP while three scaffolds, 31, 132, and 231, contained two (**Table 1**). Analysis of genomic distribution of *Z. marina* AQPs revealed a tandem duplication of ZmNIP4 family members, ZmNIP4-1 and ZmNIP4-2 located on scaffold_231.

**Table 1:** Description and distribution of aquaporins identified in *Zostera marina* genome.

				Scaffold					Protein		
Sl. No.	Gene	Phytozome ID	Gene Length (bp)	Location	Start	End	Transcript length (bp)	CDS length (bp)	Length (aa)	mw (kDa)	pI
1	ZmNIP1-1	Zosma431g00060	1224	Scaffold_431	34873	36096	906	837	278	29.52	8.62
2	ZmNIP1-2	Zosma7531g00010	642	Scaffold_7531	636	1277	642	642	213	22.52	9.99
3	ZmNIP1-3	Zosma84g00450	1202	Scaffold_84	363618	364819	879	660	219	23.81	5.9
4	ZmNIP4-1	Zosma231g00020	964	Scaffold_231	17959	18922	801	801	266	29.09	8.3
5	ZmNIP4-2	Zosma231g00030	752	Scaffold_231	29752	30503	666	666	221	24.43	8.59
6	ZmNIP5-1	Zosma22g01220	1447	Scaffold_22	939187	940633	1294	765	254	26.49	6.78
7	ZmNIP5-2	Zosma2446g00010	1044	Scaffold_2446	1	1044	891	576	192	19.96	9.04
8	ZmNIP5-3	Zosma26g01390	873	Scaffold_26	859679	860551	720	720	239	25.21	9.68
9	ZmPIP1-1	Zosma129g00200	1252	Scaffold_129	359101	360352	1172	876	291	31.39	8.3
10	ZmPIP1-2	Zosma16g00380	1100	Scaffold_16	235799	236898	708	708	235	25.55	8.22
11	ZmPIP2-1	Zosma21g01120	1348	Scaffold_21	945011	946358	1268	843	280	29.97	9.54
12	ZmPIP2-2	Zosma49g00340	892	Scaffold_49	249623	250514	819	819	272	28.97	8.45
13	ZmSIP1-1	Zosma12g00280	2031	Scaffold_12	480219	482249	1014	774	257	27.63	9.63
14	ZmSIP1-2	Zosma221g00210	1785	Scaffold_221	86562	88346	1160	726	241	26.15	7.56
15	ZmSIP2-1	Zosma132g00610	1209	Scaffold_132	414430	415638	1115	765	254	28.17	10.38
16	ZmSIP2-2	Zosma132g00640	940	Scaffold_132	420728	421667	768	768	255	28.36	10.08
17	ZmSIP2-3	Zosma29g00960	1005	Scaffold_29	581320	582324	866	816	271	29.57	9.55
18	ZmTIP1-1	Zosma24g00710	979	Scaffold_24	469609	470587	759	759	252	26.39	8.65
19	ZmTIP1-2	Zosma31g00130	846	Scaffold_31	53817	54662	756	756	251	25.66	5.09
20	ZmTIP1-3	Zosma43g00730	907	Scaffold_43	543521	544427	753	753	250	26.21	7.23
21	ZmTIP1-4	Zosma470g00020	1553	Scaffold_470	48092	49644	1397	807	268	28.24	6.96
22	ZmTIP1-5	Zosma50g01000	1332	Scaffold_50	651788	653119	1117	750	249	25.98	6.68
23	ZmTIP1-6	Zosma127g00340	839	Scaffold_127	475276	476114	759	759	252	25.88	6.13
24	ZmTIP3-1	Zosma4g01350	950	Scaffold_4	1086053	1087002	720	720	239	25.44	6.97
25	ZmTIP5-1	Zosma31g01330	884	Scaffold_31	757025	757908	813	813	270	28.24	6.93


### Phylogenetic Distribution of AQPs in *Zostera marina*

**FIGURE 1:**
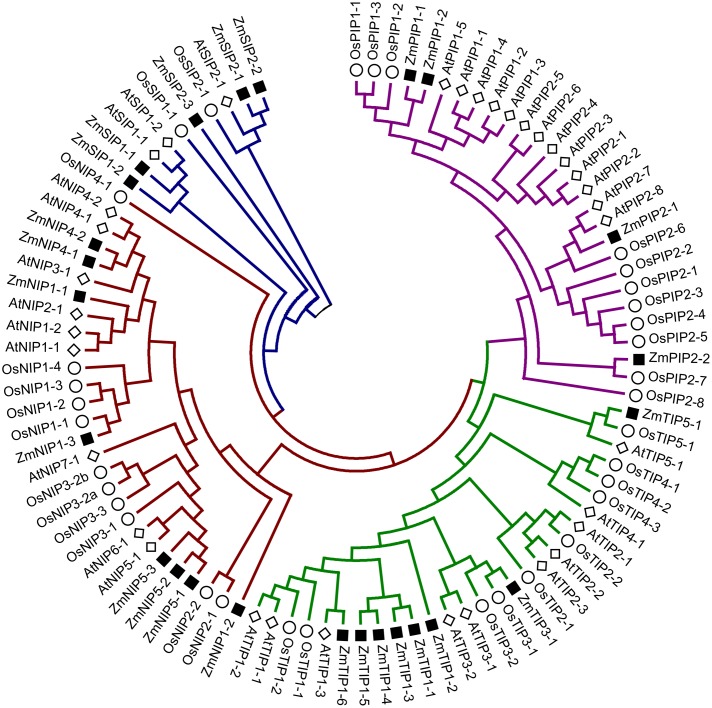
Phylogenetic tree of *Zostera marina* aquaporins (AQPs) along with rice and *Arabidopsis* AQPs representing five different groups. The genes from *Z. marina*, rice and *Arabidopsis* are preceded by the prefixes Zm, Os, and At, respectively.

**FIGURE 2:**
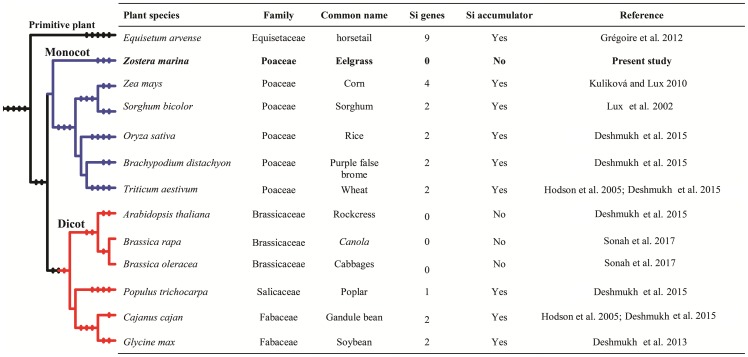
Details of silicon accumulation, and identified silicon influx transporter genes in different monocotyledon and dicotyledon plant species.

Phylogenetic tree of *Z. marina* AQPs with the known AQPs from *Arabidopsis thaliana*, and *Oryza sativa* showed four distinct clusters representing different subfamilies of AQPs (**Figure 1**). The *Z. marina* AQPs were named according to their grouping with the known AQPs, which showed 4 PIPs, 8 TIPs, 8 NIPs, and 5 SIPs. Members of the XIP family are absent from *Z. marina* genome. Within the groups formed by *Z. marina* AQPs, two major subgroups were found in ZmPIPs: ZmPIP1 and ZmPIP2. Both ZmPIP1 and ZmPIP2 are comprised of two members each. Similarly, the ZmTIPs formed five subgroups with ZmTIP1 having six members, and ZmTIP3 and ZmTIP5 having one member each. The ZmNIPs formed two distinct groups, ZmNIP1 and ZmNIP5, which are comprised of three members each, and ZmNIP4 containing two members. The NIP2 subgroup (commonly classified as NIP-III) was not found in *Z. marina* genome, a rather surprising result since the NIP-III subfamily is involved in silicon transport and has been ubiquitously reported in different monocots (**Figure 2**). ZmSIPs formed two subgroups, ZmSIP1 and ZmSIP2, represented by two and three members, respectively. BLAST search performed against non-redundant *Z. marina* nucleotide sequences at NCBI further confirmed the loss of XIPs and NIP-IIIs.

### Gene Structure, Organization and Evolution of *Zostera marina* AQPs

**FIGURE 3:**
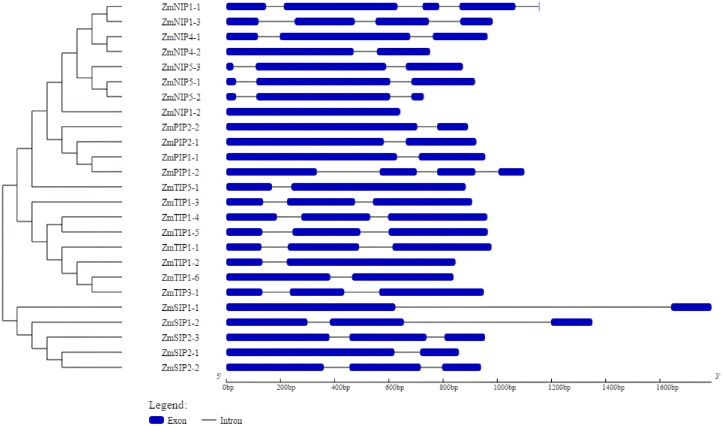
Exon–intron organization of 25 aquaporin (AQP) genes identified in *Zostera marina* genome. Graphical representation of the gene model was obtained with Gene Structure Display Server (http://gsds.cbi.pku.edu.cn/). Exons are shown as blue boxes and introns are shown as black lines. The scale at the bottom indicates length in base pairs.

The *Z. marina* AQPs showed variation in transcript (ranging from 642 to 774 bp) and gene length (ranging from 642 to 2031 bp). Exon–intron structure analysis revealed intron number variation among the AQPs contributing to the variation in gene length (**Figure 3**). The number of introns in *Z. marina* AQPs ranged from zero (ZmNIP1-2) to four (ZmNIP1-1). The lowest number of introns were observed in TIPs and SIPs with 1–2 introns followed by PIPs with 1–3, and NIPs with 0–4. Among the eight TIPs, five homologs contained two introns while three homologs harbored a single intron each. Among the NIPs, most of them (4) harbored two introns, and NIP1-2 was found to be intron-free. The SIP family members showed either one or two introns. The identified AQPs from *Z. marina* predicted proteins ranging from 192 (ZmNIP5-2) to 291 (ZmPIP1-1) amino acids.

### Characterization of NPA Motif, Transmembrane Domains and Sub-Cellular Localization – of *Z. marina* AQPs

The *Z. marina* AQPs showed amino acid differences at NPA motifs, ar/R selectivity filters and Froger’s positions (**Table 2**). Most of the AQPs contained the expected dual NPA motifs except for ZmPIP1-2, and ZmNIP1-3, which contained a single NPA motif. All members from the TIP sub-family showed conserved NPAs, while those from the SIP sub-family showed wide variation in NPA motifs compared to AQP counterparts in *Arabidopsis*. In the PIP sub-family, ZmPIP2-1 showed the substitution alanine to serine in the first NPA motif. ZmNIP1-2 showed a similar substitution. The NIP family members (ZmPIP5-1, ZmPIP5-2, and ZmPIP5-3) harbored serine in lieu of alanine in the first NPA motif and valine in lieu of alanine in the second one.

**Table 2:** Details of conserved domains, aromatic/arginine (ar/R) selectivity filter, and Froger’s residue of aquaporins in *Zostera marina* genome.

Loci	NPA (LB)	NPA (LE)		ar/R filters				Frogers residue				
				H2	H5	LE1	LE2	P1	P2	P3	P4	P5
**Plasma membrane intrinsic proteins (PIPs)**													
ZmPIP1-1	NPA	NPA		F	H	T	R	M	S	A	F	W
ZmPIP1-2		NPA		F	H	T	R		S	A	F	W
ZmPIP2-1	NPS	NPA		F	H	T	R	M	S	A	F	W
ZmPIP2-2	NPA	NPA		F	H	T	R	S	S	A	F	V
**Nodulin 26-like intrinsic proteins (NIPs)**													
ZmNIP1-1	NPA	NPA		W	V	A	R	F	T	A	Y	M
ZmNIP1-2	NPS	NPA		F	I	G	R	F	S	A	Y	L
ZmNIP1-3	NPA			W	V				F		H	F
ZmNIP4-1	NPA	NPA		W	V	A	R	L	S	S	Y	I
ZmNIP4-2	NPA	NPA		W	I	A	R	L	S	S	Y	M
ZmNIP5-1	NPS	NPV		A	I	G	R	Y	T	A	Y	L
ZmNIP5-2	NPS	NPV		A	I	G	R	Y	T			
ZmNIP5-3	NPS	NPV		A	I	G	R	Y	T	A	Y	M
**Tonoplast intrinsic proteins (TIPs)**													
ZmTIP1-1	NPA	NPA		H	I	A	V	T	A	S	Y	W
ZmTIP1-2	NPA	NPA		Y	I	G	A	T	S	A	Y	W
ZmTIP1-3	NPA	NPA		H	I	A	V	T	A	S	Y	W
ZmTIP1-4	NPA	NPA		H	I	A	M	T	S	A	Y	W
ZmTIP1-5	NPA	NPA		H	I	A	V	T	S	A	Y	W
ZmTIP1-6	NPA	NPA		H	I	A	V	T	A	A	Y	W
ZmTIP3-1	NPA	NPA		S	I	A	R		T	A	Y	W
ZmTIP5-1	NPA	NPA		Q	V	G	R	T	S	A	Y	W
**Small basic intrinsic proteins (SIPs)**													
ZmSIP1-1	NPT	NPA		L	V	P	N	M	A	A	Y	W
ZmSIP1-2	SLA	NPA		I	I	P	N	M	A	A	Y	W
ZmSIP2-1	TTL	SPA		S	D	G	K	V	F	T	N	F
ZmSIP2-2	HPL	NPA		Y	D	S	E	F	A	A	Y	V
ZmSIP2-3	NPL	NPA		A	H	G	T	F	A	A	Y	W


All the PIP sub-family members showed conserved ar/R filter residues with phenylalanine in H2, histidine at H5, threonine at LE1, and arginine at LE2. In the TIP sub-family, the H2 position of the ar/R filter consisted of histidine/tyrosine/serine/glutamine and the H5 position was comprised of isoleucine, with the exception of valine in ZmTIP5-1. The LE1 position of TIPs was occupied by glycine/alanine, while the LE2 position had four possible residues (alanine/valine/methionine/arginine). The NIP sub-family members contained phenylalanine/tryptophan/alanine (H2), valine/isoleucine (H5) alanine/glycine (LE1) and arginine (LE2). Finally, the SIP family members harbored serine/leucine/isoleucine/alanine/tyrosine (H2), isoleucine/valine/aspartic acid/histidine (H5), glycine/proline/serine (LE1), asparagine/glutamic acid/lysine/threonine (LE2).

To ascertain the expression of *Z marina* AQPs at different cellular/organellar levels, their sub-cellular localizations were predicted (Supplementary Table 4). The majority of *Z marina* PIPs were predicted to be localized in the plasma membrane. Most of NIPs (five) were targeted to the vacuole and the remaining three were predicted to be located in the plasma membrane. Among the eight TIPs, four are predicted to be located in the cytoplasm and the other ones in the plasma membrane/nucleus/chloroplast. Among SIPs, SIP1s are predicted to be in the vacuole and SIP2s in either the plasma membrane or the mitochondria.

### AQP Expression Profiling in *Zostera marina*

**FIGURE 4:**
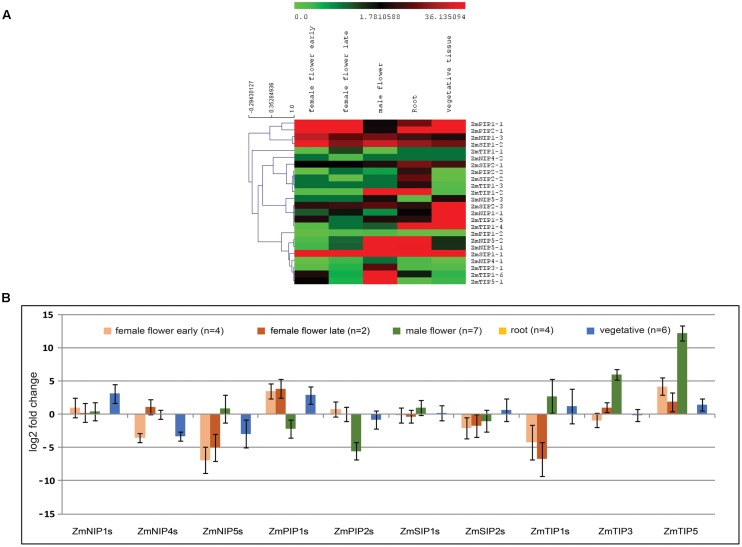
Analysis of *Zostera marina* aquaporins (AQPs) expression in different tissues using RNA-seq data (SRP056873, SRA database). **(A)** Normalized expression of AQPs in terms of reads per kilobase of transcript per million mapped reads (RPKM) showing higher levels of TIP1 and TIP5 in male flowers. **(B)** Fold change expression of aquaporin (AQP) genes in different tissues compared to roots showing similar or higher levels of expression in vegetative tissues in most AQPs, except for NIP4s and NIP5s. Bars represent standard error from the mean.

Analysis of reported RNA-seq data showed evidence of expression in 24 out of the 25 predicted *Z*. *marina* AQPs, ZmNIP1-2 being the sole absent. Family specific expression of AQPs in different tissues was calculated in terms of fold change in comparison to root tissues. The majority of AQPs showed similar or higher expression in vegetative compared to root tissues (**Figure 4A**). Among different AQPs, PIPs showed the highest expression across the different tissues analyzed. TIPs showed a particularly high expression in male flowers (**Figure 4B**).

## Discussion

Aquaporins have gained increased attention recently because of their reported pivotal role in biotic and abiotic stress tolerance (Hu et al., 2012; Liu et al., 2013; Shivaraj et al., 2017; Sonah et al., 2017). Considering the importance of eelgrass in marine environments, and its declining population associated with different stressful conditions, we were therefore interested in identifying all potential aquaporins in the species with the objective that it would become an invaluable resource to better understand its ecology and predicament.

Our genome-wide analyses led to the identification of 25 putative AQPs in *Z. marina* genome, a number inferior to reference land plants such as rice (34) (Sakurai et al., 2005; Nguyen et al., 2013) and *Arabidopsis* (35) (Johanson et al., 2001). Lineage of *Z. marina* diverged from the terrestrial monocots about 130 MYA. Its adaption to submerged conditions occasioned a loss and gain of multiple genes compared to terrestrial or floating aquatic plants (Janssen and Bremer, 2004; Olsen et al., 2016). The reduced number of AQPs in *Z. marina* compared to terrestrial plants represents likely an adaptive strategy during its evolution to a new environment. More specifically, the aquatic environment may call for a lesser need in proteins involved in water and solute acquisition through the roots.

Phylogenetic analysis of the *Z. marina* AQPs identified only four different sub-families, PIPs, TIPs, NIPs, and SIP, as observed in monocots and Brassicaceae, with the notable absence of XIP subfamily members. Interestingly, most of the dicots harbor five AQP subfamilies including XIPs (See Supplementary Table 5), which suggests a clear conservation of the absence of XIPs in monocots even following the divergence between terrestrial and aquatic plants. On the other hand, members of NIP2 (NIP-III), NIP3, TIP2, and TIP4 were not observed in *Z. marina* genome, while they are systematically found in monocots. The loss of the TIP subfamily is rather surprising since TIPs are relatively more conserved across diverse species compared to the rest of AQP subfamilies (Deshmukh et al., 2015). This likely means that TIPs have specific functions in terrestrial plants that are obsolete or redundant in eelgrass. Notwithstanding the differences in the members and number of AQPs in eelgrass, the gene structure and exon–intron organization were found to be quite similar compared to other terrestrial monocots (Danielson and Johanson, 2008). The conserved exon-intron organization is suggestive of functional redundancy across species. Since the exon–intron organization is known to be affected by gene duplication, diversification and changes in exon–intron organization may lead to changes in gene expression profile (Roy and Penny, 2007; Deshmukh et al., 2016b).

The substrate specificity of AQPs is determined by the size and hydrophobic nature of the amino acids forming the pores (Lee et al., 2005; Törnroth-Horsefield et al., 2006). All PIP family members from *Z. marina* contained a very hydrophilic ar/R selectivity filter, FHTR, a hallmark of water-transporting aquaporins, in contrast to other families. A similar ar/R selectivity filter is also observed in PIP family of aquaporins from other plant species such as *O. sativa*, *A. thaliana*, *Brassica rapa*, *Glycine Max*, and *Ricinus communis* (Johanson et al., 2001; Deshmukh et al., 2013; Zhang et al., 2013; Diehn et al., 2015; Zou et al., 2015). Among *Z. marina* TIP sub family members, TIP1s were found to have residues HIAV, YIGA, and HIAM forming a more hydrophobic ar/R filter compared to the one found in ZmTIP3 and ZmTIP5, which contained SIAR and QVGR residues, respectively. The residues present in ar/R selectivity filter of ZmTIPs were found to be similar to the TIPs from other plant species. TIPs act as functional water transporters and facilitate transport of small solutes such as NH_4_^+^, H_2_O_2_, and urea (Liu et al., 2003; Holm et al., 2005; Bienert et al., 2007). Conserved NPA motifs and ar/R filter in TIPs suggests their involvement in transport of water as well as solutes in *Z. marina*.

Members of NIP-III subfamily are known to be involved in transport of metalloids like boron (Takano et al., 2006) and silicon (Ma et al., 2006). Several studies have reported beneficial effects of Si on monocotyledonous plants. Different plant species accumulate a wide range of Si, from 0.2% or less to 10% Si on a dry weight basis (Lux et al., 2002; Hodson et al., 2005; Kuliková and Lux, 2010; Grégoire et al., 2012; Deshmukh et al., 2015). Very few studies have reported Si levels in *Z. marina* leaves; Herman et al. (1996) found a range between 0.02 and 0.66%, with higher concentrations in plant associated with higher dissolved Si in water. Interestingly, a tight positive correlation was also observed between dissolved Si levels and *Z. marina* population (Herman et al., 1996; Disney et al., 2014). On the other hand, Kamermans et al. (1999) could not establish if increase in dissolved Si levels resulted in increased biomass of *Z. marina*. This is an important feature to clarify in light of the association of declining populations of eelgrass with Si concentrations. If lower Si is indeed responsible for this situation, one must be able to establish that eelgrass benefits from Si through its uptake. Indeed, in terrestrial plants, the benefits of Si are directly correlated with a plant’s ability to absorb silicic acid. Recent studies have clearly shown that uptake of Si is mediated by aquaporins, and more specifically by NIP-IIIs containing a GSGR selectivity filter (Deshmukh and Bélanger, 2015). In the present study, we found that NIP-IIIs were completely absent from the *Z. marina* genome, which would indicate that the species is unable to uptake Si, a conclusion supported by the low Si levels found in the tissue of eelgrass. Under these conditions, it is difficult to rationalize how dissolved Si levels in water would influence eelgrass populations. Better controlled studies are thus needed to determine the role of Si, if any, on eelgrass. In any event, results from this study, namely the absence of NIP-IIIs, offer new avenues to better understand the ecology of eelgrass in the context of Si fluxes in marine environments.

Eelgrass has been extensively studied to understand the plant physiology particularly under aquatic and high saline condition (Yu, 2015; Yang et al., 2017). However, very limited attention was paid to study the involvement of AQPs in physiological processes. In *Posidonia oceanica* (Seagrass), significant role of PIPs have been observed in salinity tolerance and maintaining water balance in the leaves (Serra et al., 2013). Similarly, expression profiling performed by Cozza and Pangaro (2009) also suggested the role of AQPs in salinity and water balance in seagrass. Apart from such few reports, no significant study evaluating physiological role of AQPs has been performed in aquatic plants.

Analysis of RNA-seq data revealed higher or similar expression of different aquaporins in vegetative compared to root tissues. In terrestrial plants, in addition to providing anchorage, roots play an important role in the absorption of water and nutrients from the soil. However, in submerged aquatic plants, vegetative parts are also actively involved in uptake of water and nutrient from the surrounding environment (Barko and Smart, 1986). PIPs are known to play a central role in transport of water; additionally PIPs are known to facilitate CO_2_ diffusion in mesophyll tissue of *A. thaliana* and *Nicotiana tabacum* affecting photosynthesis (Flexas et al., 2006; Heckwolf et al., 2011). Our expression analysis showed higher expression of PIPs in different plant parts analyzed suggesting a possible role of PIPs in water transport and CO_2_ diffusion in *Z. marina*. The members of TIP1 and TIP5 family showed higher expression specifically in male flowers. Different studies in *A. thaliana* have shown pollen specific accumulation of TIP family members (TIP1;3 and TIP5;1), which are expected to be involved in pollen maturation and germination (Soto et al., 2008; Wudick et al., 2014). Similar expression pattern of homologs of these TIP members in *Z. marina* indicates their conserved role in pollen development affecting reproduction.

## Conclusion

Genome-wide analysis of AQPs performed in the first fully sequenced marine angiosperm, *Z. marina* revealed several salient features. It has shown loss of AQPs in *Z. marina* compared to reference land plants such as rice and *Arabidopsis.* The reduced number of AQPs in *Z. marina* compared to terrestrial plants represents likely an adaptive strategy during its evolution to a new environment. The *Z. marina* AQPs formed only four different sub-families as observed in monocots and Brassicaceae, with the notable absence of XIP subfamily members. We also found absence of NIP-III members from the *Z. marina* genome, which would indicate that the species is unable to uptake Si, a conclusion supported by the low Si levels reported in the tissue of eelgrass. The absence of NIP-IIIs in *Z. marina*, offer new avenues to understand the ecology of eelgrass in the context of the role of Si in marine environments. The higher expression for most of the AQPs in shoots compared to roots observed with RNA-seq data suggests, as one could expect, a predominant role of vegetative tissues in uptake of water and nutrient from the surrounding environment. The members of TIP1 and TIP5 family showed higher expression specifically in male flowers as observed in *A. thaliana* indicating their probable role in pollen development affecting reproduction. The identification, classification, and expression of AQPs performed in the present study will be helpful for enhancing our knowledge of distribution and evolution of AQPs in aquatic plant species.

## Author Contributions

SS, RD, and HS compiled the data, performed analysis, and wrote first draft of the MS. HS and JB performed expression analysis. RB planned the study, drew the conclusions and contributed to the writing of the manuscript.

## Conflict of Interest Statement

The authors declare that the research was conducted in the absence of any commercial or financial relationships that could be construed as a potential conflict of interest.

## Footnotes

1 https://phytozome.jgi.doe.gov/pz/portal.html

2 http://www.cbs.dtu.dk

3 http://harrier.nagahama-i-bio.ac.jp

4 http://mev.tm4.org/#/welcome
