# Supplementary material for: Understanding Aquaporin Transport System in Eelgrass (Zostera marina L.), an Aquatic Plant Species
Source: Front Plant Sci. 2017 Aug 3;8:1334. doi: 10.3389/fpls.2017.01334 (PMC5541012; doi:10.3389/fpls.2017.01334)
Supplement: Supplementary file 2 [file Table_1.DOCX]

**Supplementary table 1.** Details of Blastp results representing hits showing highest bit score for the respective queries from other plant genome.

| **Sl. No.** | **Query** | **Blast Hit** | **e-value** | **Bit Score** |
| --- | --- | --- | --- | --- |
| 1 | OsNIP1-1 | Zosma431g00060 | 6.00E-92 | 279 |
| 2 | GmNIP1-6 | Zosma7531g00010 | 3.00E-46 | 214 |
| 3 | OsNIP1-1 | Zosma84g00450 | 3.00E-76 | 220 |
| 4 | AtNIP4-2 | Zosma231g00020 | 3.00E-81 | 267 |
| 5 | AtNIP4-1 | Zosma231g00030 | 2.00E-68 | 222 |
| 6 | AtNIP5-1 | Zosma22g01220 | 2.00E-88 | 255 |
| 7 | AtNIP5-1 | Zosma2446g00010 | 3.00E-75 | 192 |
| 8 | AtNIP5-1 | Zosma26g01390 | 2.00E-91 | 240 |
| 9 | AtPIP1-3 | Zosma129g00200 | 1.00E-144 | 292 |
| 10 | AtPIP1-3 | Zosma16g00380 | 2.00E-108 | 236 |
| 11 | AtPIP2-8 | Zosma21g01120 | 5.00E-135 | 281 |
| 12 | OsPIP2-2 | Zosma49g00340 | 5.00E-86 | 273 |
| 13 | OsSIP1-1 | Zosma12g00280 | 6.00E-71 | 258 |
| 14 | GmSIP1-4 | Zosma221g00210 | 6.00E-74 | 242 |
| 15 | AtSIP2-1 | Zosma132g00610 | 3.00E-26 | 255 |
| 16 | AtSIP2-1 | Zosma132g00640 | 3.00E-41 | 256 |
| 17 | GmSIP2-1 | Zosma29g00960 | 1.00E-60 | 272 |
| 18 | GmTIP1-2 | Zosma24g00710 | 1.00E-92 | 253 |
| 19 | AtTIP1-1 | Zosma31g00130 | 2.00E-83 | 252 |
| 20 | AtTIP1-1 | Zosma43g00730 | 2.00E-97 | 251 |
| 21 | OsTIP1-1 | Zosma470g00020 | 2.00E-103 | 269 |
| 22 | OsTIP1-1 | Zosma50g01000 | 3.00E-109 | 250 |
| 23 | AtTIP1-3 | Zosma127g00340 | 6.00E-106 | 253 |
| 24 | OsTIP3-1 | Zosma4g01350 | 8.00E-88 | 240 |
| 25 | OsTIP5-1 | Zosma31g01330 | 9.00E-76 | 271 |
