# Supplementary material for: Understanding Aquaporin Transport System in Eelgrass (Zostera marina L.), an Aquatic Plant Species
Source: Front Plant Sci. 2017 Aug 3;8:1334. doi: 10.3389/fpls.2017.01334 (PMC5541012; doi:10.3389/fpls.2017.01334)
Supplement: Supplementary file 3 [file Table_2.DOCX]

**Supplementary table 2.** Conserved domain analysis of *Z. marina* AQPs using CDD tool (NCBI)

| **Sl. No.** | **Query** | **PSSM-ID** | **E-Value** | **Bitscore** | **Accession** | **Short name** | **Superfamily** |
| --- | --- | --- | --- | --- | --- | --- | --- |
| 1 | ZmNIP1-1 | 294134 | 4.26E-106 | 308.874 | cl00200 | MIP superfamily | - |
| 2 | ZmNIP1-2 | 294134 | 4.51E-52 | 166.275 | cl00200 | MIP superfamily | - |
| 3 | ZmNIP1-3 | 294134 | 6.20E-85 | 252.635 | cl00200 | MIP superfamily | - |
| 4 | ZmNIP4-1 | 294134 | 1.11E-108 | 314.54 | cl00200 | MIP superfamily | - |
| 5 | ZmNIP4-2 | 294134 | 1.46E-90 | 266.39 | cl00200 | MIP superfamily | - |
| 6 | ZmNIP5-1 | 177663 | 2.19E-124 | 354.168 | PLN00026 | PLN00026 | cl00200 |
| 7 | ZmNIP5-2 | 177663 | 2.40E-102 | 295.618 | PLN00026 | PLN00026 | cl00200 |
| 8 | ZmNIP5-3 | 177663 | 1.81E-127 | 361.487 | PLN00026 | PLN00026 | cl00200 |
| 9 | ZmPIP1-1 | 278651 | 3.78E-99 | 288.832 | pfam00230 | MIP | cl00200 |
| 10 | ZmPIP1-2 | 294134 | 2.40E-63 | 195.614 | cl00200 | MIP superfamily | - |
| 11 | ZmPIP2-1 | 278651 | 1.05E-99 | 289.988 | pfam00230 | MIP | cl00200 |
| 12 | ZmPIP2-2 | 294134 | 2.43E-74 | 225.274 | cl00200 | MIP superfamily | - |
| 13 | ZmSIP1-1 | 294134 | 2.84E-17 | 76.9086 | cl00200 | MIP superfamily | - |
| 14 | ZmSIP1-2 | 294134 | 1.06E-14 | 69.5898 | cl00200 | MIP superfamily | - |
| 15 | ZmSIP2-2 | 294134 | 7.36E-08 | 50.3298 | cl00200 | MIP superfamily | - |
| 16 | ZmSIP2-3 | 294134 | 7.71E-11 | 59.1894 | cl00200 | MIP superfamily | - |
| 17 | ZmTIP1-1 | 177664 | 1.60E-117 | 334.832 | PLN00027 | PLN00027 | cl00200 |
| 18 | ZmTIP1-2 | 294134 | 4.19E-98 | 285.912 | cl00200 | MIP superfamily | - |
| 19 | ZmTIP1-3 | 177664 | 8.13E-133 | 373.738 | PLN00027 | PLN00027 | cl00200 |
| 20 | ZmTIP1-4 | 177664 | 6.03E-135 | 379.901 | PLN00027 | PLN00027 | cl00200 |
| 21 | ZmTIP1-5 | 177664 | 1.03E-139 | 391.071 | PLN00027 | PLN00027 | cl00200 |
| 22 | ZmTIP1-6 | 177664 | 1.58E-123 | 350.24 | PLN00027 | PLN00027 | cl00200 |
| 23 | ZmTIP3-1 | 294134 | 1.50E-93 | 273.586 | cl00200 | MIP superfamily | - |
| 24 | ZmTIP5-1 | 294134 | 1.99E-84 | 252.131 | cl00200 | MIP superfamily | - |
