# Supplementary material for: Understanding Aquaporin Transport System in Eelgrass (Zostera marina L.), an Aquatic Plant Species
Source: Front Plant Sci. 2017 Aug 3;8:1334. doi: 10.3389/fpls.2017.01334 (PMC5541012; doi:10.3389/fpls.2017.01334)
Supplement: Supplementary file 4 [file Table_3.DOCX]

**Supplementary table 3.** Transmembrane domains in AQPs identified from *Z. marina* using TMHMM and SOSUI servers

|  | **TMHMM** | | | |  | **SOSUI** | |  |
| --- | --- | --- | --- | --- | --- | --- | --- | --- |
| **gene** | **Length** | **ExpAA** | **First 60** | **predHel** |  | **protein type** | **helix** | **Max TM** |
| ZmNIP1-1 | 278 | 129.3 | 9.9 | 6 |  | MP | 6 | 6 |
| ZmNIP1-2 | 213 | 132.43 | 42.26 | 6 |  | MP | 6 | 6 |
| ZmNIP1-3 | 219 | 107.16 | 19.07 | 5 |  | MP | 5 | 5 |
| ZmNIP4-1 | 266 | 129.3 | 19.94 | 5 |  | MP | 6 | 6 |
| ZmNIP4-2 | 221 | 124.13 | 36.16 | 6 |  | MP | 6 | 6 |
| ZmNIP5-1 | 254 | 131.38 | 35.16 | 6 |  | MP | 6 | 6 |
| ZmNIP5-2 | 192 | 109.4 | 35.16 | 5 |  | MP | 5 | 5 |
| ZmNIP5-3 | 239 | 126.26 | 38.58 | 6 |  | MP | 6 | 6 |
| ZmPIP1-1 | 291 | 129.04 | 6.65 | 6 |  | MP | 6 | 6 |
| ZmPIP1-2 | 235 | 108.31 | 6.48 | 5 |  | MP | 5 | 5 |
| ZmPIP2-1 | 280 | 129.83 | 21.57 | 6 |  | MP | 5 | 6 |
| ZmPIP2-2 | 272 | 127.63 | 22.71 | 6 |  | MP | 6 | 6 |
| ZmSIP1-1 | 257 | 131.55 | 22.83 | 6 |  | MP | 6 | 6 |
| ZmSIP1-2 | 241 | 144.95 | 41.31 | 7 |  | MP | 5 | 7 |
| ZmSIP2-1 | 254 | 112.83 | 13.71 | 5 |  | MP | 6 | 6 |
| ZmSIP2-2 | 255 | 94.91 | 19.3 | 5 |  | SP | NONE | 5 |
| ZmSIP2-3 | 271 | 140.64 | 21.58 | 7 |  | MP | 5 | 7 |
| ZmTIP1-1 | 252 | 140.49 | 24.44 | 6 |  | MP | 6 | 6 |
| ZmTIP1-2 | 251 | 134.6 | 23.98 | 6 |  | MP | 6 | 6 |
| ZmTIP1-3 | 250 | 143.63 | 24.61 | 7 |  | MP | 6 | 7 |
| ZmTIP1-4 | 268 | 146.56 | 12.14 | 6 |  | MP | 6 | 6 |
| ZmTIP1-5 | 249 | 139.59 | 24.47 | 6 |  | MP | 6 | 6 |
| ZmTIP1-6 | 252 | 151.81 | 25.43 | 7 |  | MP | 7 | 7 |
| ZmTIP3-1 | 239 | 132.18 | 26.89 | 6 |  | MP | 6 | 6 |
| ZmTIP5-1 | 270 | 129.03 | 21.29 | 5 |  | MP | 8 | 8 |

ExpAA: The expected number of amino acids in transmembrane helices.

First60: The expected number of amino acids in transmembrane helices in the first 60 amino acids of the protein.

MP: Membrane protein

TMH: The number of predicted transmembrane helices.

MAX TMH: The maximum number among the TMH predicted by TMHMM and SOSUI
