# Supplementary material for: Understanding Aquaporin Transport System in Eelgrass (Zostera marina L.), an Aquatic Plant Species
Source: Front Plant Sci. 2017 Aug 3;8:1334. doi: 10.3389/fpls.2017.01334 (PMC5541012; doi:10.3389/fpls.2017.01334)
Supplement: Supplementary file 5 [file Table_4.DOCX]

**Supplementary table 4.** Details of predicted sub-cellular location of *Z. marina* AQPs identified by using Wolfpsort, Cello and TargetP servers

|  | **Wolfsport** | **Cello** | **TragetP** |
| --- | --- | --- | --- |
| ZmNIP1-1 | PlasmaMembrane | PlasmaMembrane | _ |
| ZmNIP1-2 | vacuole | PlasmaMembrane | Secretory |
| ZmNIP1-3 | PlasmaMembrane | PlasmaMembrane | _ |
| ZmNIP4-1 | vacuole | PlasmaMembrane | _ |
| ZmNIP4-2 | PlasmaMembrane | PlasmaMembrane | Secretory |
| ZmNIP5-1 | vacuole | PlasmaMembrane | _ |
| ZmNIP5-2 | vacuole | PlasmaMembrane | _ |
| ZmNIP5-3 | vacuole | PlasmaMembrane | Secretory |
| ZmPIP1-1 | PlasmaMembrane | PlasmaMembrane | _ |
| ZmPIP1-2 | PlasmaMembrane | PlasmaMembrane | _ |
| ZmPIP2-1 | PlasmaMembrane | PlasmaMembrane | _ |
| ZmPIP2-2 | PlasmaMembrane | PlasmaMembrane | _ |
| ZmSIP1-1 | vacuole | PlasmaMembrane | _ |
| ZmSIP1-2 | vacuole | PlasmaMembrane | Secretory |
| ZmSIP2-1 | PlasmaMembrane | PlasmaMembrane | Mitochondra |
| ZmSIP2-2 | mitochondria | PlasmaMembrane | _ |
| ZmSIP2-3 | PlasmaMembrane | PlasmaMembrane | _ |
| ZmTIP1-1 | cytoplasm | PlasmaMembrane | _ |
| ZmTIP1-2 | nucleus | PlasmaMembrane | _ |
| ZmTIP1-3 | cytoplasm | PlasmaMembrane | Mitochondra |
| ZmTIP1-4 | cytoplasm | PlasmaMembrane | _ |
| ZmTIP1-5 | cytoplasm | PlasmaMembrane | _ |
| ZmTIP1-6 | PlasmaMembrane | PlasmaMembrane | _ |
| ZmTIP3-1 | chloroplast | PlasmaMembrane | _ |
| ZmTIP5-1 | chloroplast | PlasmaMembrane | _ |

^1^<http://wolfpsort.org/>, ^2^<http://cello.life.nctu.edu.tw>, ^3^[www.cbs.dtu.dk/services/TargetP](http://www.cbs.dtu.dk/services/TargetP)
