# Supplementary material for: Understanding Aquaporin Transport System in Eelgrass (Zostera marina L.), an Aquatic Plant Species
Source: Front Plant Sci. 2017 Aug 3;8:1334. doi: 10.3389/fpls.2017.01334 (PMC5541012; doi:10.3389/fpls.2017.01334)
Supplement: Supplementary file 6 [file Table_5.DOCX]

**Supplementary table 5.** Distribution of number of aquaporins in different plant species

| **Plant Species** |  | **AQP** | **PIP** | **TIP** | **NIP** | **XIP** | **SIP** | **Reference** |
| --- | --- | --- | --- | --- | --- | --- | --- | --- |
| *Zostera marina* | Monocot  (model aquatic plant) | 25 | 4 | 8 | 8 | 0 | 5 |  |
| *Physcomitrella patens* | Primitive plant | 23 | 9 | 4 | 5 | 2 | 2 | (Danielson and Johanson, 2008) |
| *Selaginella moellendorfﬁi* | Primitive plant | 19 | 3 | 3 | 8 | 3 | 1 | (Anderberg et al., 2014; Deshmukh et al., 2015) |
| *Picea abies* | Primitive plant | 39 | 18 | 6 | 13 | 0 | 2 | (Deshmukh et al., 2015) |
| *Musa acuminata* | monocot | 51 | 21 | 18 | 9 | 0 | 3 | (Deshmukh et al., 2015) |
| *Oryza sativa* | Monocot  (model plant) | 34 | 11 | 10 | 11 | 0 | 2 | (Sakurai et al., 2005) |
| *Brachypodium distachyon* | monocot | 32 | 11 | 10 | 9 | 0 | 2 | (Deshmukh et al., 2015) |
| *Sorghum bicolor* | monocot | 40 | 14 | 13 | 10 | 0 | 3 | (Deshmukh et al., 2015) |
| *Zea mays* | monocot | 43 | 10 | 13 | 13 | 0 | 7 | (Deshmukh et al., 2015) |
| *Setaria italica* | monocot | 50 | 16 | 16 | 15 | 0 | 3 | (Deshmukh et al., 2015) |
| *Elaeis guineensis* | monocot | 30 | 9 | 10 | 9 | 0 | 2 | (Deshmukh et al., 2015) |
| *Hordeum vulgare L* | monocot | 22 | 11 | 7 | 4 | 0 | 2 | (Hove et al., 2015) |
| *Phyllostachys edulis* | monocot | 26 | 10 | 6 | 8 | 0 | 2 | (Sun et al., 2016) |
| *Arabidopsis thaliana* | Dicot  (model plant) | 35 | 13 | 10 | 9 | 0 | 3 | (Quigley et al., 2001) |
| *Arabidopsis lyrata* | Dicot | 39 | 14 | 12 | 10 | 0 | 3 | (Deshmukh et al., 2015) |
| *Brassica rapa* | Dicot | 59 | 22 | 16 | 15 | 0 | 6 | (Deshmukh et al., 2015) |
| *Brassica oleracea* | Dicot | 67 | 25 | 19 | 17 | 0 | 6 | (Deshmukh et al., 2015) |
| *Carica papaya* | Dicot | 28 | 10 | 7 | 7 | 2 | 2 | (Deshmukh et al., 2015) |
| *Citrus sinensis* | Dicot | 34 | 11 | 9 | 8 | 3 | 3 | (Deshmukh et al., 2015) |
| *Citrus clementina* | Dicot | 37 | 14 | 10 | 9 | 1 | 3 | (Deshmukh et al., 2015) |
| *Vitis vinifera* | Dicot | 30 | 9 | 9 | 9 | 2 | 1 | (Deshmukh et al., 2015) |
| *Glycine max* | Dicot | 72 | 22 | 23 | 17 | 2 | 8 | (Deshmukh et al., 2013) |
| *Cajanus cajan* | Dicot | 40 | 12 | 13 | 10 | 1 | 4 | (Deshmukh et al., 2015) |
| *Fragaria vesca* | Dicot | 39 | 10 | 9 | 14 | 2 | 4 | (Deshmukh et al., 2015) |
| *Prunus persica* | Dicot | 29 | 7 | 8 | 9 | 2 | 3 | (Deshmukh et al., 2015) |
| *Ricinus communis* | Dicot | 36 | 10 | 9 | 8 | 5 | 4 | (Deshmukh et al., 2015) |
| *Populus trichocarpa* | Dicot | 58 | 15 | 18 | 11 | 7 | 7 | (Gupta and Sankararamakrishnan, 2009) |
| *Solanum tuberosum* | Dicot | 44 | 15 | 11 | 11 | 5 | 2 | (Deshmukh et al., 2015) |
| *Solanum lycopersicum* | Dicot | 44 | 14 | 10 | 11 | 6 | 3 | (Deshmukh et al., 2015) |
| *Phaseolus vulgaris* | Dicot | 41 | 12 | 13 | 10 | 2 | 4 | (Ariani and Gepts, 2015) |
| *Hevea brasiliensis Muell. Arg.* | Dicot | 51 | 15 | 17 | 9 | 6 | 4 | (Zou et al., 2015) |
| *Jatropha curcas* | Dicot | 32 | 9 | 9 | 8 | 2 | 4 | (Zou et al., 2016) |
| **Total 31 plant species** |  | **1224** | **402** | **348** | **316** | **53** | **105** |  |
